# One-step and two-step qPCR assays for CAPRV2023: development and application in full-cycle epidemiological surveillance of golden pompano

**DOI:** 10.3389/fvets.2025.1620997

**Published:** 2025-07-31

**Authors:** Heng Sun, Bissih Fred, Haoyu Wang, Jie Huang, Zihao Wu, Dandan Wu, Yishan Lu, Jichang Jian, Yucong Huang

**Affiliations:** 1 Guangdong Provincial Key Laboratory of Aquatic Animal Disease Control and Healthy Culture, Fisheries College of Guangdong Ocean University, Zhanjiang, China; 2 Key Laboratory of Control for Disease of Aquatic Animals of Guangdong Higher Education Institutes, Fisheries College, Guangdong Ocean University, Zhanjiang, China

**Keywords:** *Carpione rhabdovirus*, CAPRV2023, *Trachinotus ovatus*, TaqMan quantitative PCR, one-step qPCR, viruses in water samples

## Abstract

*Carpione rhabdovirus* strain 2023 (CAPRV2023) has recently emerged as a significant pathogen responsible for substantial mortality in farmed golden pompano (*Trachinotus ovatus*) across China, threatening the sustainability of the aquaculture industry. To address the urgent need for rapid and accurate diagnostics, we developed two TaqMan probe-based quantitative PCR (qPCR) assays targeting the viral G protein gene: a two-step qPCR assay and a one-step qPCR assay. The newly developed two-step qPCR assay demonstrated excellent performance, with a detection limit of 2 copies/μL and an amplification efficiency of 104.7%. The intra- and inter-assay coefficients of variation (CVs) ranged from 0.23 to 0.95% and 0.28 to 1.95%, respectively. The one-step qPCR assay further simplified the detection workflow by integrating reverse transcription and amplification into a single closed-tube reaction. It achieved a detection limit of 15 copies/μL, with a high amplification efficiency (102.8%) and excellent repeatability (CV = 0.81%). Specificity tests demonstrated that no cross-reactivity was observed with other aquatic pathogens. Extensive validation across clinical and environmental samples revealed that the two-step and one-step qPCR assays consistently exhibited higher detection sensitivity than conventional PCR. Their reliable performance across multiple geographic locations and sampling periods confirmed robust diagnostic reliability, indicating strong tolerance to potential viral mutations and excellent adaptability to diverse aquaculture environments. In addition, the two qPCR assays enabled accurate quantification of viral loads in aquaculture water samples concentrated via ultrafiltration, demonstrating their value in environmental surveillance. Overall, both the two-step and one-step qPCR assays represent robust, sensitive, and field-adaptable diagnostic platforms, with extensive applications in disease surveillance, early outbreak warning, pathogenesis studies, and aquaculture biosecurity.

## 1 Introduction

*Carpione rhabdovirus* (CAPRV) is a newly identified member of the fish rhabdovirus family, distinguished from other species within the genus *Novirhabdovirus* by its unique full genome and serological characteristics. CAPRV strain LG1988 was initially discovered in the carpione *Salmo trutta* carpio in Italy in 1987 and caused high mortality in the fry (1). Since then, no further cases of CARPV infection have been reported. In 2023, a new CAPRV strain CAPRV2023 linked to high mortality was identified in golden pompano (*Trachinotus ovatus*) farmed in offshore cages in China, resulting in substantial economic losses. Notably, the full genome similarity between CAPRV2023 and CAPRV583 is only 81.32% (2). According to the species classification criteria of *Novirhabdovirus* established by the International Committee on Taxonomy of Viruses (ICTV), the newly revised “Taxonomy of Rhabdoviruses Infecting Fish and Marine Mammals” categorizes it as an unclassified virus within this genus (3).

Currently, the newly emerging infectious diseases lack effective prevention and control measures, owing to the absence of antiviral drugs and vaccines. Thus, developing highly precise and sensitive molecular assays for the early and accurate detection of CAPRV2023 is essential for effective prevention and control of its transmission (2). In addition to traditional necropsy and pathological examinations, a variety of diagnostic methods such as cell culture, *in situ* hybridization, electron microscopy, and RT-PCR followed by agarose gel electrophoresis have been developed to detect CAPRV2023 (2). However, these techniques require suitable laboratory environments. In recent years, quantitative PCR (qPCR) instruments have become increasingly affordable and compact, which greatly enhances their accessibility and convenience. Based on these advancements, the development of qPCR technology aligns well with the current market demands and holds significant application potential in both laboratory settings and front-line aquaculture operations (4). To further enhance the detection specificity and sensitivity and accurately quantify CAPRV2023 in clinical samples, a SYBR Green I quantitative PCR method was established in our previous study (2). However, because SYBR Green I can bind to all double-stranded DNA, it can lead to non-specific amplifications, resulting in false positives or inaccurate quantifications (5–7).

TaqMan probe-based qPCR is widely recognized as the gold standard for detecting aquatic pathogens owing to its high sensitivity, specificity, and rapid detection capabilities. For instance, two-step qPCR assays have been developed for quantitative detection and monitoring of Tilapia lake virus (TiLV) (8) and viral hemorrhagic septicemia virus (VHSV) (9–12) in aquaculture. However, no studies have reported the application of TaqMan probe-based qPCR for CAPRV2023 detection in field samples.

This study introduces a TaqMan two-step qPCR assay and a TaqMan one-step qPCR assay targeting the G protein gene for the detection of CAPRV2023, following thorough assessment of its sensitivity, repeatability, and specificity. The two-step and one-step qPCR assays exhibited superior efficiency, specificity, and sensitivity compared with conventional PCR methods. Furthermore, this one-step qPCR assay enabled the quantification of viral loads in both naturally and experimentally infected golden pompano and in water samples from CAPRV2023-associated disease outbreaks.

## 2 Materials and methods

### 2.1 Clinical samples and ethics statement

Golden pompanos were randomly sampled from 27 offshore cage farms across China between May 2024 and January 2025. A total of 87 clinical samples (including at least three fish per site) were obtained for analysis. The CAPRV2023 strain was isolated from infected golden pompanos and preserved in the laboratory (2). Fathead minnow (FHM) cells preserved at the Guangdong Provincial Key Laboratory of Aquatic Animal Disease Control and Healthy Culture were cultivated in Leibovitz's L-15 medium (Biosharp, Beijing, China) supplemented with 10% fetal bovine serum and maintained at 28°C. The virus isolation and culture procedures followed the methods described by Sun et al. (2). The CAPRV2023 was introduced into confluent FHM cell monolayers, and those exhibiting cytopathic effects were collected and stored at −80°C. All animal experiments were conducted strictly based on the recommendations in the “Guide for the Care and Use of Laboratory Animals set by the National Institutes of Health”.

### 2.2 Extraction of total RNA and synthesis of cDNA

RNA was isolated from ~20 ± 0.2 mg of spleen tissues collected from golden pompano and infected cell cultures, and cDNA were prepared as described by Sun et al. (2). The spleen was chosen for RNA extraction and viral detection because previous studies have shown that it harbors the highest viral load in CAPRV2023-infected golden pompanos, thereby providing optimal sensitivity for molecular assays (2).

### 2.3 Primer and probe design for the TaqMan qPCR

Specific primers and probes were designed based on the conserved G protein-coding sequences (CDS) of 12 CARPB2023 strains originating from diverse geographical regions and isolation time points (data not shown).

CAPRV-G-COPY was specifically developed to target a conserved region shared by both CAPRV2023 and CAPRV581, allowing detection of both strains. In contrast, other primers and probes were designed specifically for CAPRV2023, due to notable sequence divergence between the two strains outside of the conserved region.

The sequences of the three primer sets and their expected product sizes are shown in Table 1. To evaluate specificity, all primer and probe sequences were compared against the NCBI database using BLAST. The TaqMan probes were synthesized by Shanghai Sangon Co., Ltd (China), and labeled with 6-carboxyfluorescein (FAM) at the 5′ end and Black Hole Quencher 1 (BHQ1) at the 3′ end.

**Table 1:** Detail of primers and probes used in this study.

**Primer**	**Sequence**	**Product**
CAPRV-Q1F	5′-CCAGAACCTGTTTGCGCTTG-3′	190
CAPRV-Q1R	5′-AGTCCCTATTCACCACCCAGA-3′	
CAPRV-Q2F	5′-CGAGGACGACTTTGGTTATCT3′	118
CAPRV-Q2R	5′-CAGAAGGTAGGAGGAGACTTTG3′	
CAPRV-Q3F	5′-AATGTAGTCTGGGTGGTGAATAG3′	111
CAPRV-Q3R	5′-CGCAACCGCCTTTATGATTG3′	
CAPRV-PROBE1	5′6-FAM-CTCACAGATAATGTGGCGGCAAAGAGGGAG-3′BHQ1	-
CAPRV-PROBE2	5′6-FAM-GGAATGCCTTGAGGCTCACAATGAGAT-3′BHQ1	-
CAPRV-PROBE3	5′6-FAM-CAGCAACCTATCCCACCAAGAACACT-3′BHQ1	-
CAPRV-G-COPY-F	5′-CGCAACCGCCTTTATGATTG3′	740
CAPRV-G-COPY-R	5′-GTCCCCTTCCTGGGTGATGAGG3′	
CAPRV-SYBR-F	5′-CAACCTATCCCACCAAGAACACT3′	152
CAPRV-SYBR-F	5′-TGTCCTGATCCATTGTTCTCCAG3′	

### 2.4 Construction of plasmid for standard quantification

A 740-bp fragment specific to CAPRV2023 was amplified using the primers CAPRV-740F and CAPRV-740R and cloned into the pCE3 Blunt Vector (Vazyme). The recombinant plasmid preparation followed the method described by Sun et al. (2). The plasmid concentration was measured with a NanoDrop™ One/OneC microspectrophotometer (Thermo Fisher Scientific, Waltham, MA, USA), yielding 136 ng/μL, which corresponds to approximately 4.98 × 10^10^ copies/μL. The sequence of the inserted fragment was verified by Sanger sequencing to confirm correct insertion and orientation.

### 2.5 Establishment and optimization of the TaqMan qPCR conditions

PCR amplification was performed using the LightCycler 96 qPCR Detection System (Roche, Stockholm, Sweden). Primer and probe concentrations and annealing temperature were optimized using a fixed template concentration of 10^5^ copies. The basic TaqMan qPCR protocol is as follows: an initial denaturation at 95°C for 60 s, followed by 40 cycles of 95°C for 10 s, gradient annealing from 51°C to 59°C for 30 s.

Before optimization, three set of qPCR primer-probe combinations were screened to identify those with the highest sensitivity (data not shown). The CAPRV2023-Q1F/Q1R-QPROBE1 set was selected as the optimal primer combination for further optimization. To determine the optimal primer concentration, the primer concentration was set within the range of 0.2–1.0 μM, and the PCR was performed at a fixed probe concentration of 62.5 nM. Similarly, probe concentrations were optimized within a range of 62.5–250 nM. The optimal annealing temperature was determined through qPCR analysis within the range of 51–59°C. The optimal primer concentration, probe concentration, and temperature were defined as those yielding the lowest threshold cycle (Ct) and the highest fluorescence intensity. These optimized parameters were used in all subsequent experiments.

### 2.6 Calibration standard curve of the two-step qPCR assay

A series of ten-fold serial dilutions of the extracted plasmid, ranging from 10^9^ to 2 copies/μL, was prepared to establish the standard curve for the two-step qPCR detection of CAPRV2023, following the method described by Sun et al. (2) A standard curve was generated by plotting the logarithm of copy number on the y-axis against the cycle threshold (Ct) on the x-axis, followed by linear regression analysis.

### 2.7 Sensitivity, specificity, repeatability, and reproducibility of the two-step qPCR

RNA was extracted from viral pathogens, including VHSV, *Siniperca chuatsi* rhabdovirus (SCRV), spring viremia of carp virus (SVCV), infectious hematopoietic necrosis virus (IHNV), nervous necrosis virus (NNV), and TiLV, and reverse-transcribed into cDNA. Additionally, DNA was extracted from infectious spleen and kidney necrosis virus (ISKNV) and Singapore grouper iridovirus (SGIV). The genomic DNA of bacterial strains such as *Streptococcus dysgalactiae, Vibrio alginolyticus, Streptococcus agalactiae, Streptococcus iniae, Lactococcus garvieae, Vibrio harveyi*, and golden pompano were also extracted for specificity assessment. The specificity of the assay was verified using cDNA and DNA isolated from common pathogens. cDNA from CAPRV2023-infected golden pompano was used as the positive control, whereas cDNA from healthy golden pompano and double-distilled water served as negative controls. Each experiment was performed in triplicate.

The repeatability of the two-step qPCR assay was evaluated by performing inter- and intra-assay tests using a 10-fold serial dilution of the standard plasmid (ranging from 10^9^–10^1^ copies/μL). Each inter- and intra-assay was conducted in triplicate across three independent runs. The coefficients of variation (CVs) were calculated based on the mean Ct values from each assay to assess repeatability.

### 2.8 Sensitivity of conventional PCR using the qPCR primer

The sensitivity of conventional PCR was evaluated using the qPCR primers with plasmid template concentrations ranging from 10^6^-10^1^ copies. The PCR program was initiated with a denaturation step at 95 °C for 5 min, followed by 40 amplification cycles consisting of denaturation at 95 °C for 30 s and annealing at 55 °C for 30 s. The samples were then separated on a 2% agarose gel for 20 min, and the target bands were visualized using a gel imaging system (Bio-Rad, Hercules, CA, USA).

### 2.9 One-step qPCR for CAPRV2023

#### 2.9.1 Standard RNA samples

RNA of CAPRV2023-infected from FHM cell was extracted, reverse transcribed and quantified using the two-step qPCR in this study. The Ct value was 15.74, which was equal to the copy numbers of 10^7.31^. This sample was diluted to the appropriate concentration for further use.

#### 2.9.2 One-step qPCR

The one-step qPCR was conducted based on the optimized conditions established for the two-step qPCR assay. The protocol comprised a reverse transcription step at 55 °C for 6 min, followed by subsequent procedures as described in Section 2.5.

#### 2.9.3 Sensitivity and standard curve of the one-step qPCR

To evaluate the detection sensitivity of the CAPRV2023 one-step qPCR assay, a serial dilution of standard RNA samples was prepared. The standard RNA was diluted in a fold gradient series (ranging from 15 to 3 × 10^7^ copies/μL), and each dilution was tested in triplicate. The limit of detection (LOD) was defined as the lowest RNA concentration that produced a reproducible Ct value ≤ 40, accompanied by a distinct amplification curve.

#### 2.9.4 Repeatability of the one-step qPCR

To evaluate the reproducibility of the one-step qPCR assay, a single sample was repeatedly tested 40 times following the protocol described in Section 2.9.2. The mean Ct value, CV%, and standard deviations (SDs) were calculated to assess the assay's repeatability.

#### 2.9.5 Specificity of the one-step qPCR

The specificity of the assay was evaluated using RNA and DNA extracted from common pathogens, following the protocol described in Section 2.7. Samples were considered positive if the Ct value was ≤ 40 and exhibited a distinct amplification curve.

### 2.10 Evaluation of clinical samples using three kinds of PCR techniques

#### 2.10.1 Detection of clinical samples

To assess the distribution and viral load of CAPRV2023 during the outbreak, water and spleen tissue samples were collected from 87 golden pompano culture cages located across central and western Guangdong, eastern Guangxi, and Hainan provinces between June 2024 and January 2025. The fish had body lengths ranging from 7.45 to 45.29 cm. A total of 87 RNA samples extracted from spleen tissues were analyzed using TaqMan one-step qPCR, two-step qPCR, and conventional PCR methods (2). Diagnostic sensitivity and specificity were determined according to the guidelines outlined in the WOAH Manual of Diagnostic Tests for Aquatic Animals (13).

A 100 mL water sample was collected from each of the 85 golden pompano aquaculture cages affected by CAPRV2023. The water samples were passed through a 0.22 μm polycarbonate membrane, effectively removing insoluble impurities and the majority of bacteria. The filtered water was subsequently concentrated using protein ultrafiltration tubes with a 10 kDa molecular weight cutoff (MWCO) using centrifugation at 3,300 × *g* for 30 min at 4°C. This concentration process was repeated until the volume was reduced to approximately 100 μL to achieve viral enrichment. RNA extraction and cDNA synthesis were performed as described in Section 2.2. Each sample was tested in triplicate, and each qPCR assay was performed in triplicate.

FHM cell culture analysis confirmed the absence of false negatives and false positives, further validating the reliability and accuracy of the CAPRV2023 two-step qPCR method for CAPRV2023 diagnosis in spleen and environmental samples.

#### 2.10.2 Viral loads in tissues

The newly developed CAPRV2023 two-step and one-step qPCR assays were used to quantify CAPRV2023 viral loads in clinical golden pompano tissue samples. RNA extraction and cDNA synthesis were conducted as described in Section 2.2. Each sample was assayed in triplicate, and duplicate qPCR were performed. Viral copy numbers in the tissues were calculated by extrapolating the average Ct values to a standard curve (2).

### 2.11 Statistical analysis

Statistical analyses were performed using GraphPad Prism 10, with chi-square tests used to evaluate the differences in positive detection rates among the three diagnostic methods (conventional PCR, one-step qPCR, and two-step qPCR). Statistical significance was set at *p* < 0.05.

To assess the agreement between the two qPCR methods, a Bland-Altman analysis was performed by plotting the differences between paired measurements against their means. The mean difference, or bias, reflects any systematic discrepancies between the methods, while the 95% limits of agreement [mean ± 1.96 × standard deviation (SD)] defined the range within which 95% of the differences were expected to fall. If most data points lay within these limits, it suggested a strong agreement between the methods; large deviations from the limits may indicate inconsistencies.

## 3 Results

### 3.1 Condition optimization of the TaqMan qPCR

**Figure 1:**
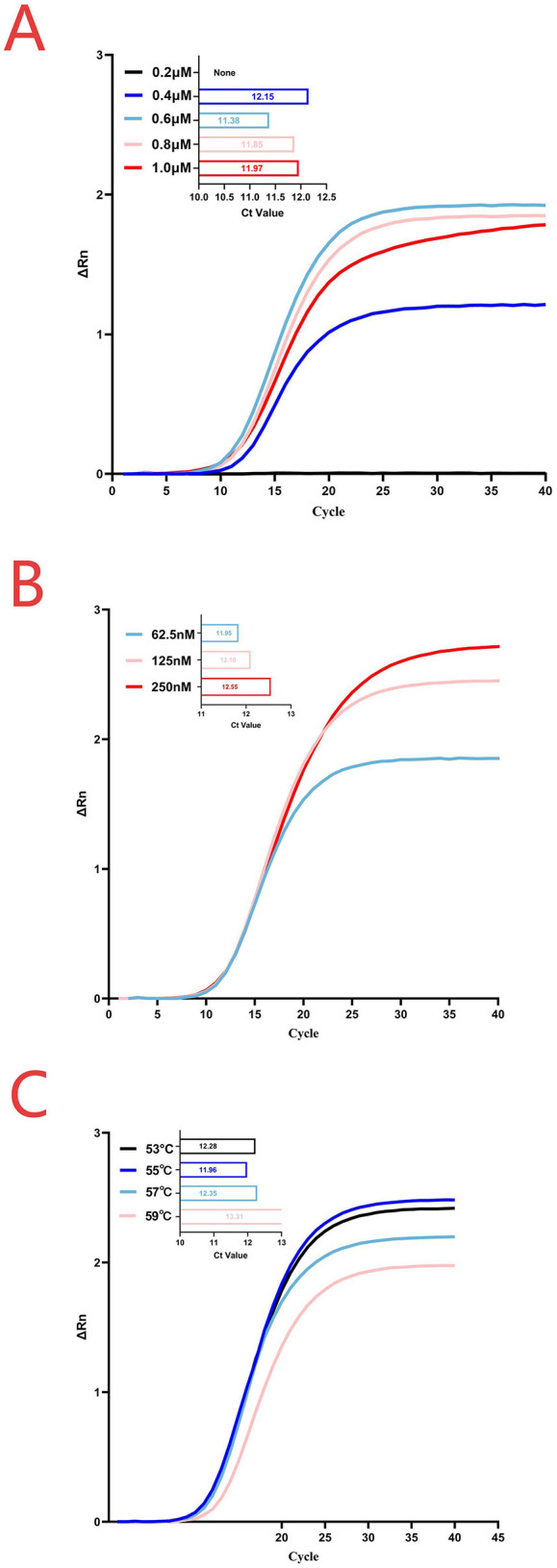
Reaction conditions for the two-step qPCR assay. **(A)** Primer concentration optimization was conducted at an annealing temperature of 55°C with a probe concentration of 62.5 nM. **(B)** Probe concentration optimization was performed using a primer concentration of 0.6 μM and an annealing temperature of 55°C. **(C)** Annealing temperature optimization was carried out using the optimal concentrations determined in the previous steps, with a primer concentration of 0.6 μM and a probe concentration of 125 nM.

The concentrations of the probe and primers were optimized through a series of experiments using various primer-probe combinations. As shown in Figure 1A, a concentration of 0.6 μM for both forward and reverse primers in the two-step qPCR produced the lowest Ct value and highest fluorescence intensity. When the probe concentrations were 62.5, 125, and 250 nM, the mean Ct values obtained from the two-step qPCR assays were 11.98, 12.15, and 12.58, respectively. According to the Roche software algorithm, the Ct values were determined to be one-tenth of the maximum fluorescence. At a probe concentration of 62.5 nM, extremely low-copy samples occasionally showed amplification curves. However, lower fluorescence led the software to identify them as negative. Therefore, 125 nM was selected as the optimal probe concentration (Figure 1B). Similarly, the optimum annealing temperature for the CAPRV2023 two-step qPCR was found to be 55°C (Figure 1A).

### 3.2 Calibration standard curve of the two-step qPCR assay

**Figure 2:**
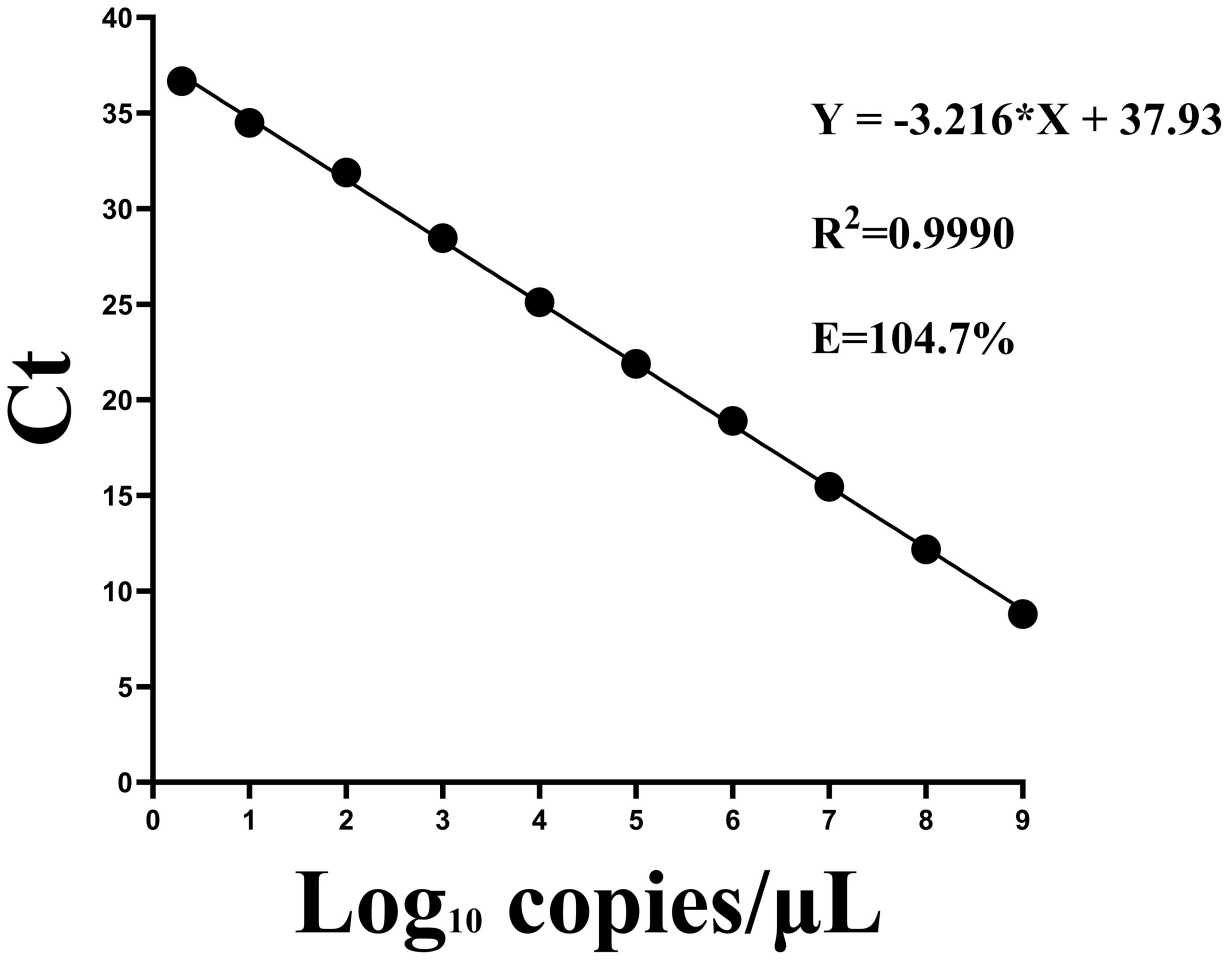
Standard curves for the two-step qPCR assay using fold serial dilutions of plasmids containing the CAPRV2023 G protein gene.

A standard curve for the two-step qPCR assay was successfully generated using serial dilutions of plasmids, ranging from 10^9^-2 copies/μL (2). A strong linear correlation was observed between template concentration and Ct values. The regression equation for the standard curve was Y = −3.216X + 37.93, where X represented the log of the viral copy number. The correlation coefficient (R^2^) was 0.9990 and the amplification efficiency was 104.7% (Figure 2). A Ct value of 40 was established as the cutoff threshold for determining positive samples. Although the standard curve was linear and quantifiable only down to 2 copies/μL (about Ct=36.5), this Ct value represents the mean of nine technical replicates using a standard RNA sample with a known nominal concentration of 2 copies/μL. Due to inherent variability at very low template concentrations, this experimentally derived value may differ slightly from theoretical estimates based on the regression equation. The cut-off was extended to 40 to maximize sensitivity for detecting low viral loads near the limit of detection. Signals >40 were deemed unreliable based on background noise analysis, ensuring high specificity and reliability.

### 3.3 Sensitivity, specificity, repeatability, and reproducibility of the two-step qPCR

**Figure 3:**
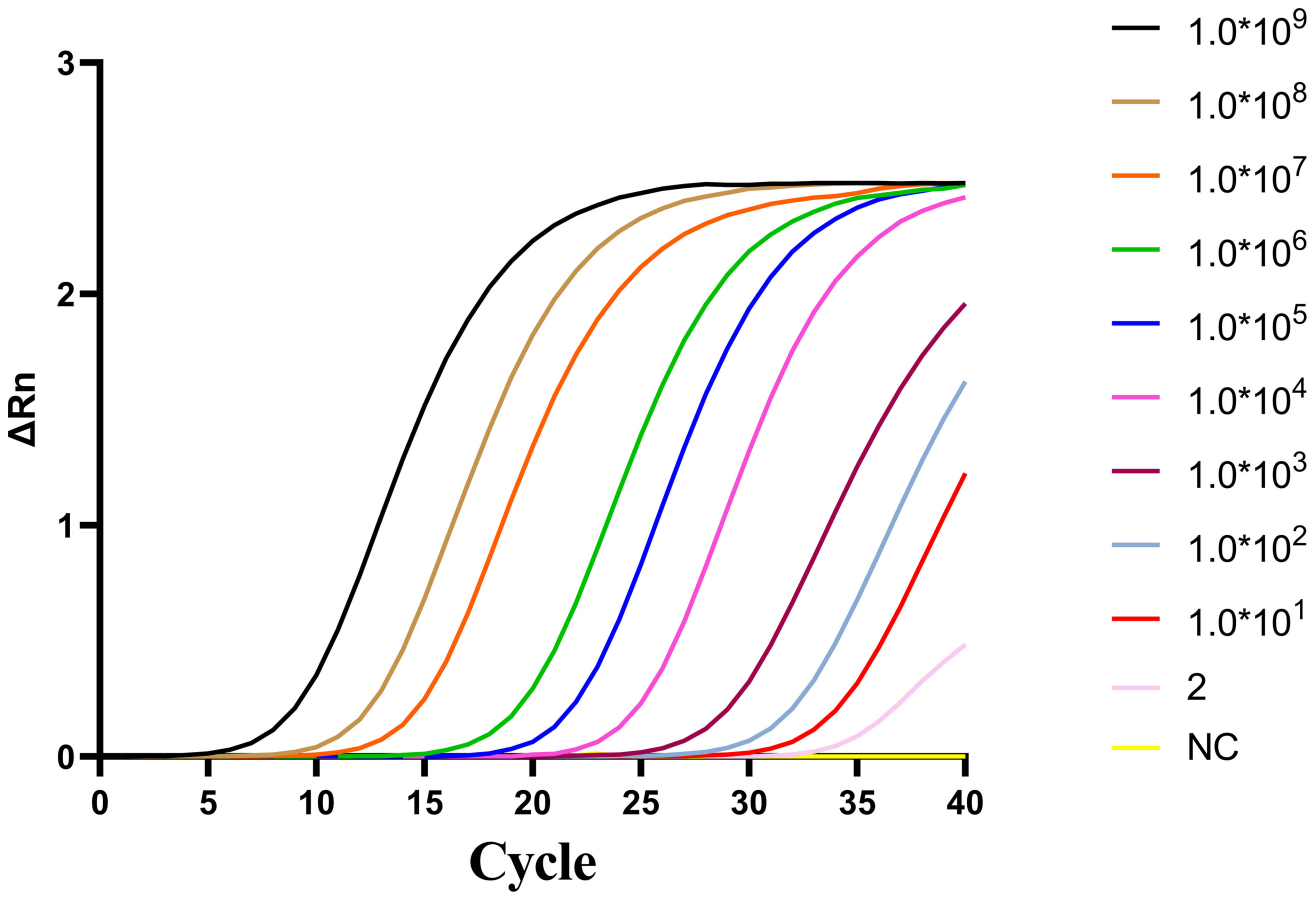
Amplification curves for the two-step qPCR assay using fold serial dilutions of plasmids containing the CAPRV2023 G protein gene.

**Figure 4:**
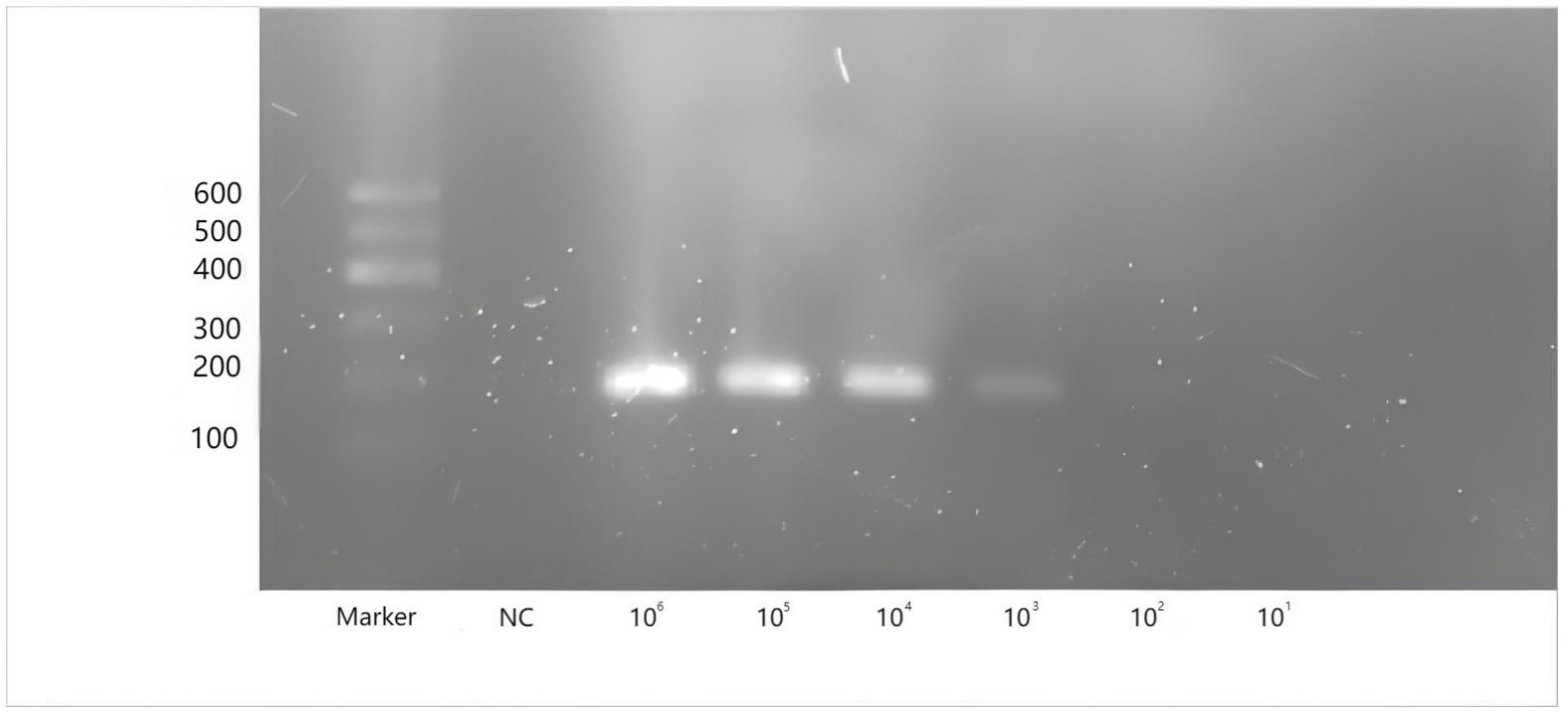
Amplification of conventional PCR for CAPRV2023 with plasmids containing G protein gene of CAPRV2023 at concentrations ranging from 10^6^ to10^1^ copies/μl. Lane Marker: DL 600bp DNA marker, Lane NC: negative control.

To evaluate the sensitivity of two-step qPCR assay was using standard plasmids were used at concentrations ranging from 10^9^-2 copies/μL. As shown in Figure 3. The newly developed two-step qPCR assay achieved an LOD of 2 copies/μL, representing a 500-fold improvement over conventional PCR (10^3^ copies/μL) (Figure 4).

**Figure 5:**
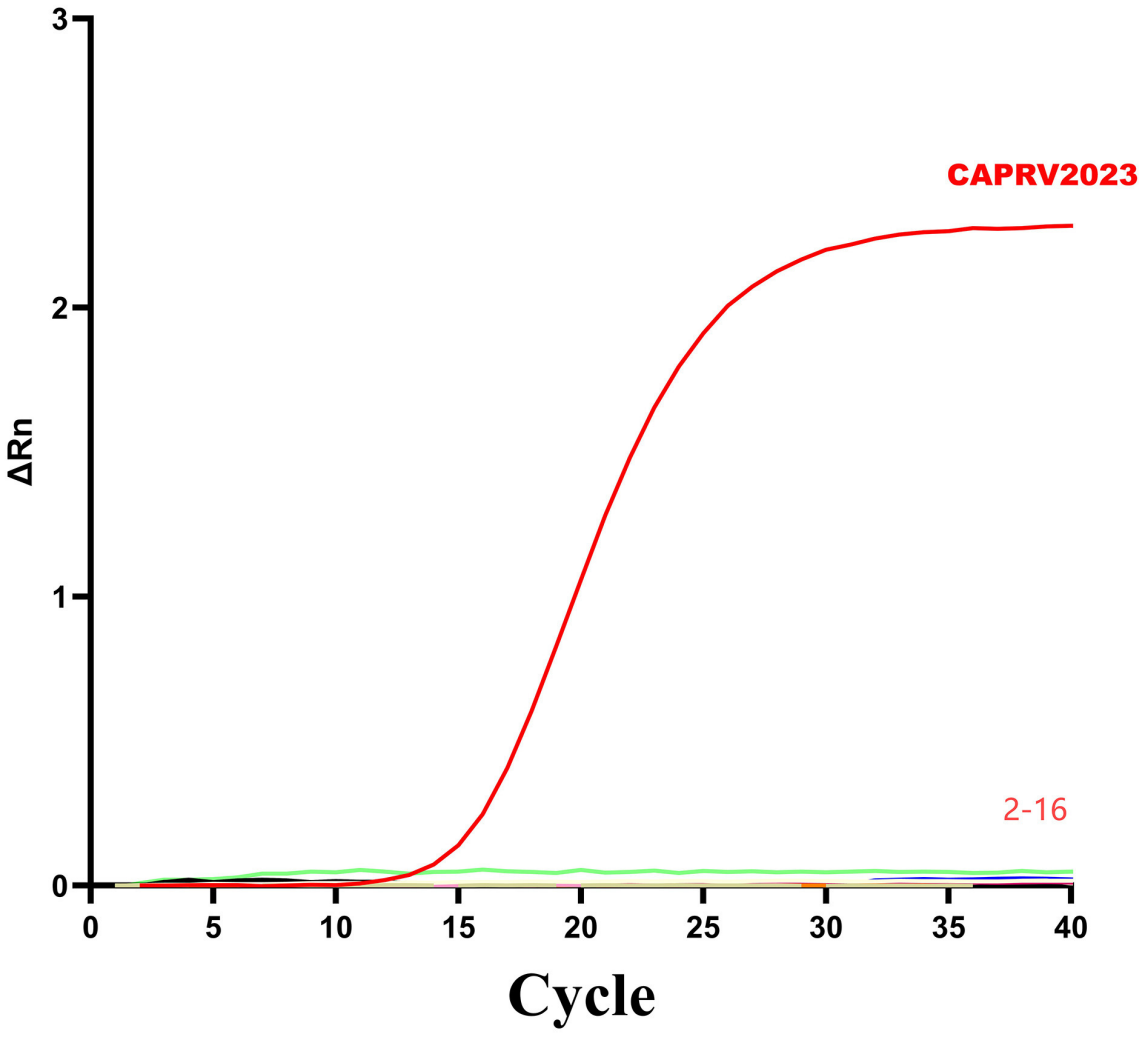
Amplification curve of the two-step qPCR assay for CAPRV2023. The assays were tested against DNA and cDNA samples: (1) CAPRV2023, (2) Negative control, (3) ISKNV, (4) SGIV, (5) TiLV, (6) SCRV, (7) NNV, (8) SVCV, (9) VHSV, (10) *V. alginolyticus*, (11) *S. dysgalactiae*, (12) *S. iniae*, (13) *L. garvieae*, (14) *S. agalactiae*, (15) *V. harveyi*, (16) Golden pompano genomic DNA.

To assess the specificity, the assay was tested against genomic DNA or cDNA from 15 common pathogens. As shown in Figure 5, amplification curves were generated only for CAPRV2023. No amplification reactions were observed for other pathogens.

The repeatability of the inter- and intra-assay reproducibility of the two-step qPCR assays was assessed by measuring the CVs of the Ct values of independent PCR reactions. The intra-assay CVs ranged from 0.23 to 0.95% (< 5%), with mean Ct values from 8.70 to 36.53 and SDs from 0.02 to 0.45 (Table 2). The inter-assay CVs varied from 0.28 to 1.95%, with mean values ranging from 8.73 to 36.63 and SDs from 0.09 to 0.45. These results confirmed the strong specificity, high sensitivity, and good repeatability and reproducibility of the newly developed two-step qPCR assay.

**Table 2:** The reproducibility of the CAPRV2023 two-step qPCR assay using standard plasmid dilutions ranging from 10^9^ to 2 copies/μL.

**Concentration of standard plasmid (copies/μl)**	**Intra-assay Mean ±SD**	**Intra-assay CV (%)**	**Inter-assay Mean ±SD**	**Inter-assay CV (%)**
10^9^	8.70 ± 0.02	0.23	8.73 ± 0.17	1.95
10^8^	12.34 ± 0.05	0.41	12.28 ± 0.13	1.06
10^7^	15.50 ± 0.03	0.42	15.53 ± 0.14	0.90
10^6^	18.86 ± 0.18	0.95	18.80 ± 0.27	1.44
10^5^	21.78 ± 0.06	0.28	21.80 ± 0.19	0.87
10^4^	24.85 ± 0.13	0.52	24.82 ± 0.28	1.13
10^3^	28.43 ± 0.08	0.27	28.48 ± 0.15	0.53
10^2^	31.83 ± 0.14	0.44	31.85 ± 0.09	0.28
10^1^	34.27 ± 0.22	0.64	34.38 ± 0.26	0.76
2	36.63 ± 0.29	0.81	36.71 ± 0.45	1.23

### 3.4 Diagnostic Performance of the one-step qPCR

**Figure 6:**
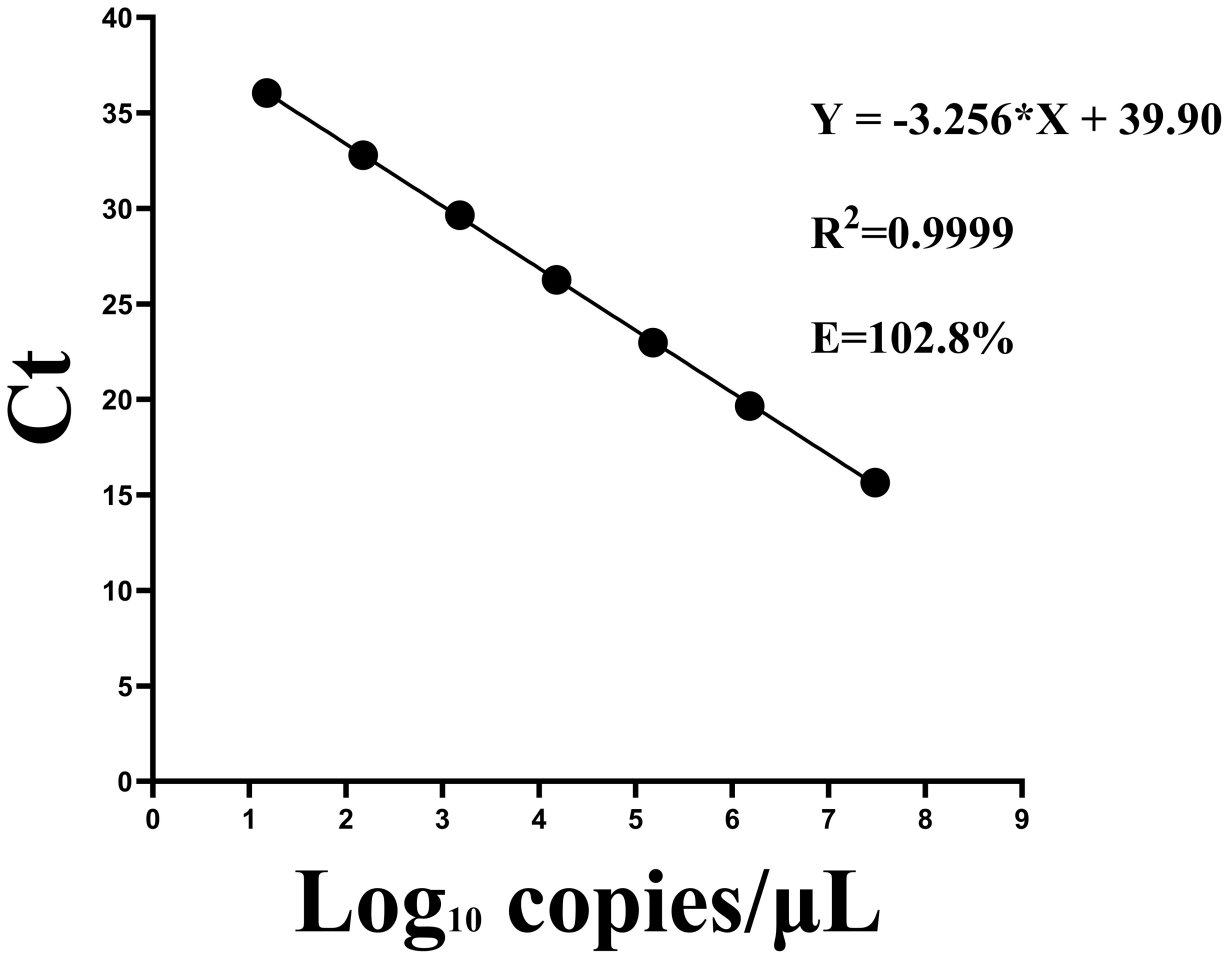
Standard curves for the one-step qPCR assay using serial dilutions of standard RNA sample containing the CAPRV2023.

A strong linear correlation was observed between template RNA concentration and Ct values. The regression equation for the standard curve was Y=-3.256X + 39.90, where X represented the log of the viral copy number. The correlation coefficient (R^2^) was 0.9999 and the amplification efficiency was 102.8% (Figure 6). Similarly, a Ct value of 40 was established as the cutoff threshold for determining positive samples.

**Figure 7:**
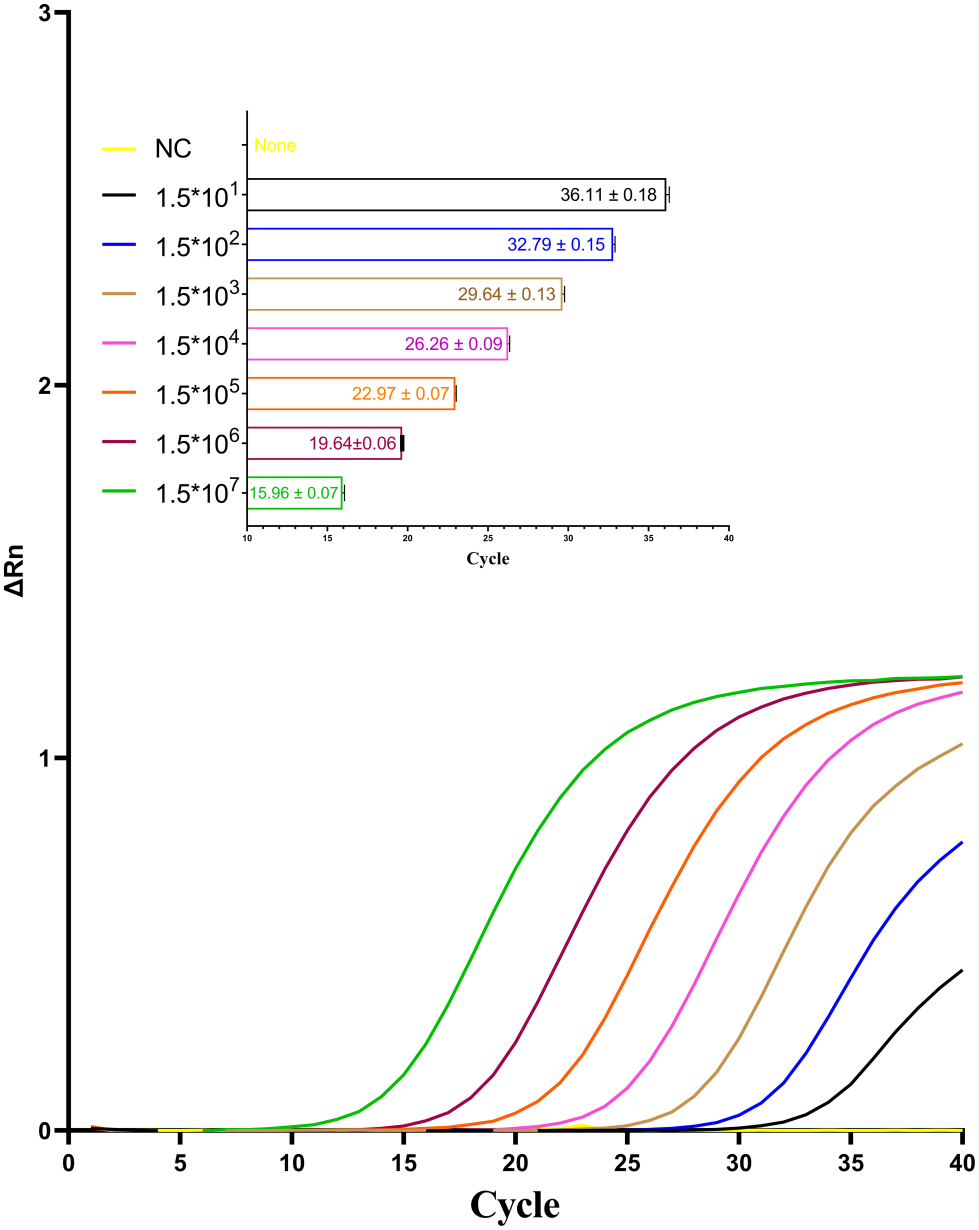
Amplification curves for the one-step qPCR assay targeting CAPRV2023 were generated using serial dilutions of standard RNA sample containing the CAPRV2023.

The sensitivity of the optimized one-step qPCR assay was evaluated using a series of standard RNA samples with concentrations ranging from 1.5 to 3 × 107 copies/μL. As shown in Figure 7, the detection limit of the one-step qPCR for CAPRV2023 RNA determined to be as low as 15 copies/μL.

**Figure 8:**
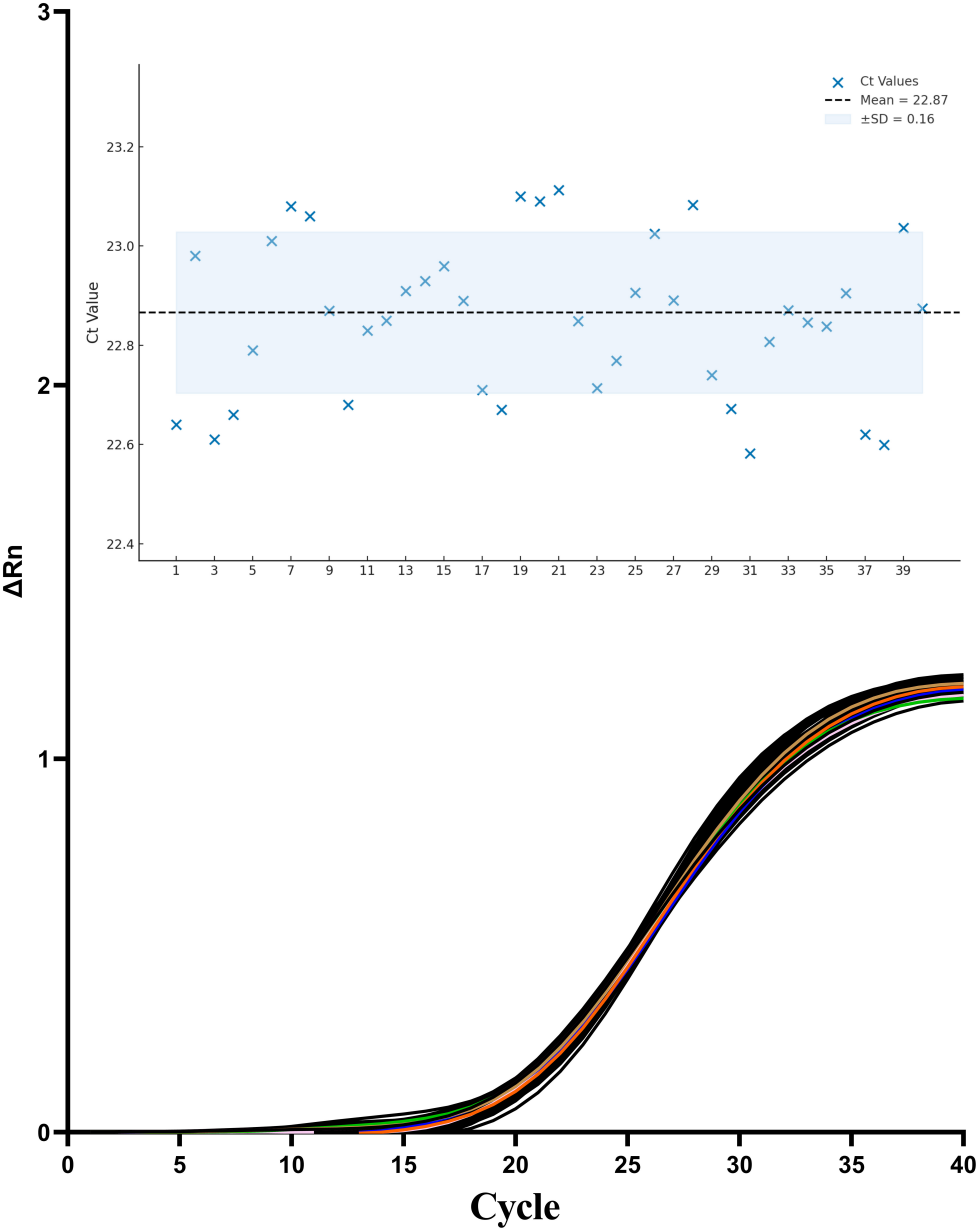
Amplification curves from 40 independent replicates using the same standard RNA sample containing CAPRV2023.

The repeatability and reproducibility of the TaqMan one-step qPCR assay were evaluated by calculating the CVs of Ct values derived from 40 independent PCR reactions using the same RNA sample. The assay yielded a CV of 0.81%(< 5%), with a mean Ct value of 22.87 ± 0.16 (Figure 8). These results demonstrate excellent repeatability and reproducibility of the newly developed one-step qPCR assay.

**Figure 9:**
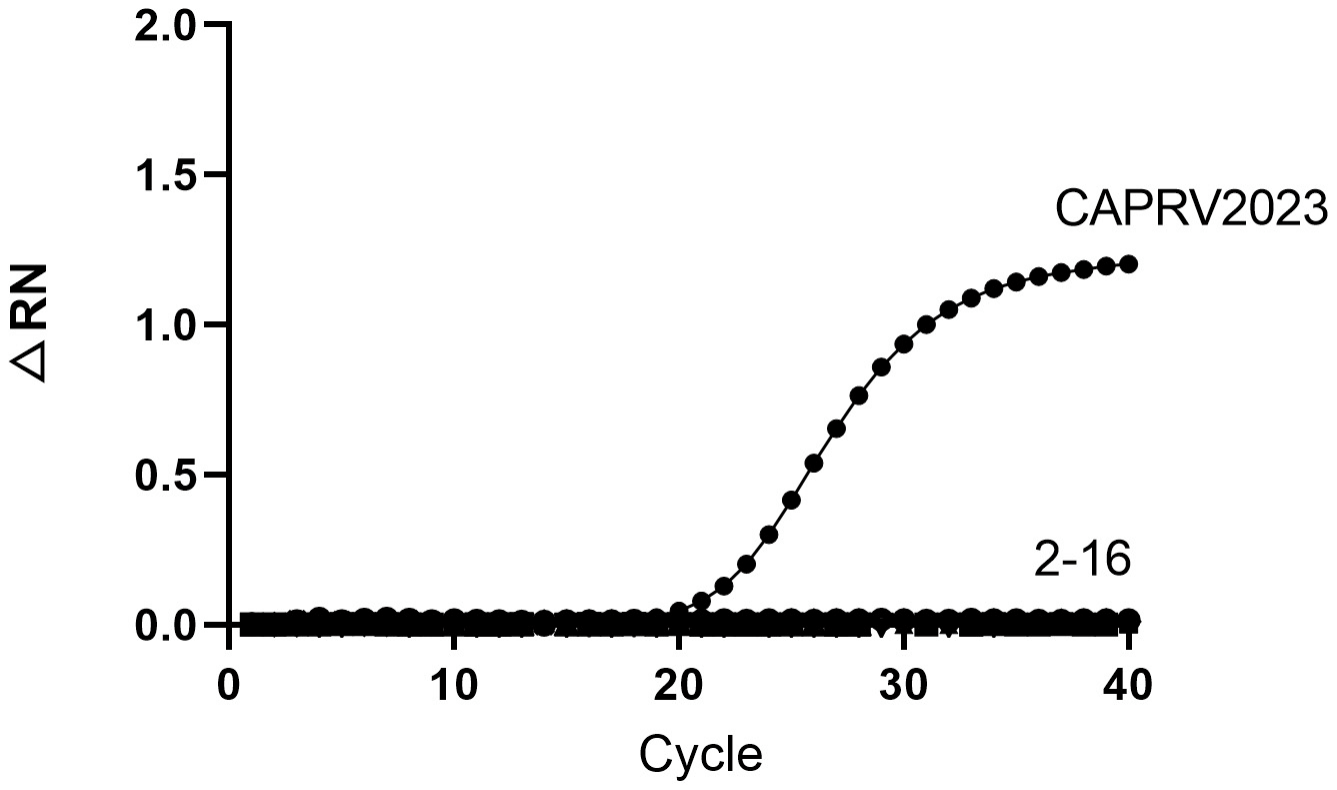
Amplification curve of the one-step qPCR assay for CAPRV2023. The assays were tested against DNA and RNA samples from infected fish tissues: (1) CAPRV2023, (2) Negative control, (3) ISKNV, (4) SGIV, (5) TiLV, (6) SCRV, (7) NNV, (8) SVCV, (9) VHSV, (10) *V. alginolyticus*, (11) *S. dysgalactiae*, (12) *S. iniae*, (13) *L. garvieae*, (14) *S. agalactiae*, (15) *V. harveyi*, (16) Golden pompano genomic DNA.

To assess the specificity, the assay was tested against genomic DNA or RNA from 15 common pathogens. As shown in Figure 9, amplification curves were generated only for CAPRV2023. No amplification reactions were observed for other pathogens.

### 3.5 Application of the two-step and one-step qPCR assays

#### 3.5.1 Detection of clinical samples

**Figure 10:**
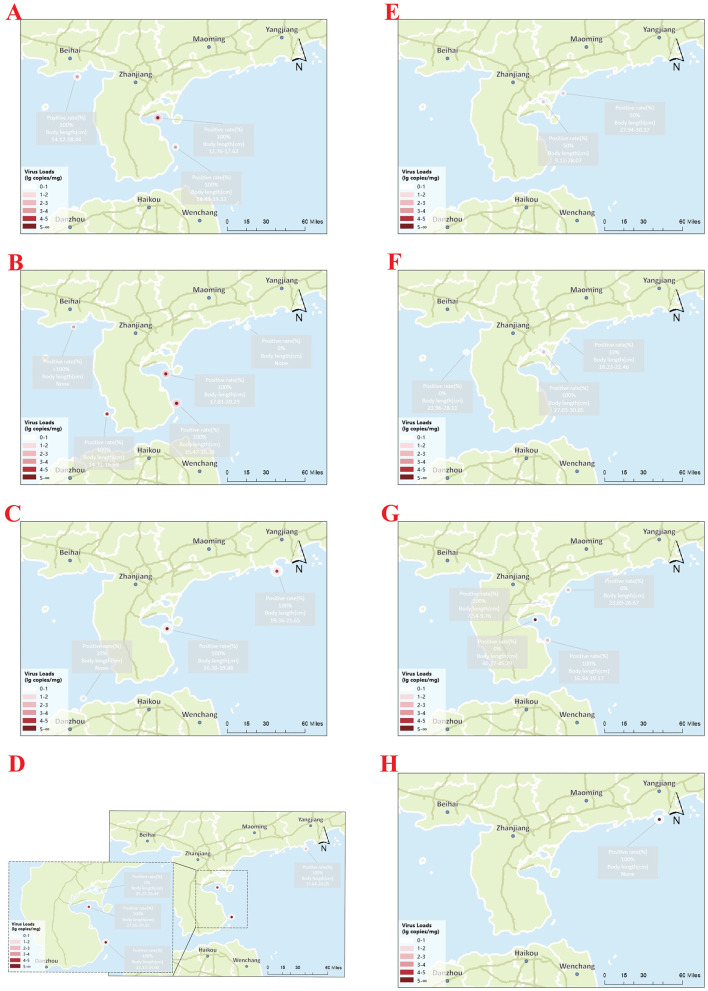
Spatial distribution and viral loads of CAPRV2023 in cage-farmed golden pompano and corresponding aquaculture water samples at different locations in the South China Sea over a complete farming cycle. Each site contains two markers: a large dot representing the viral load (lg copies/mg) in aquaculture water samples and a small dot representing the viral load in the spleen tissues of sampled fish. Color intensity reflects the viral copy number, as indicated in the legend. Panels **(A–H)** correspond to eight consecutive monthly sampling points from June 2024 to January 2025. The associated positivity rates (%) and fish body lengths (cm) are indicated for each location.

As shown in Figure 10, positive detection rates varied across sampling months and regions from June 2024 to January 2025 using the two-step and one-step qPCR. Specifically, the qPCR-positive rates for water and tissue samples were 77.63% and 66.2%, respectively, which were substantially higher than those detected using conventional PCR (19.4% and 41.4%, respectively). Detailed results are provided in Supplementary Table 1.

**Figure 11:**
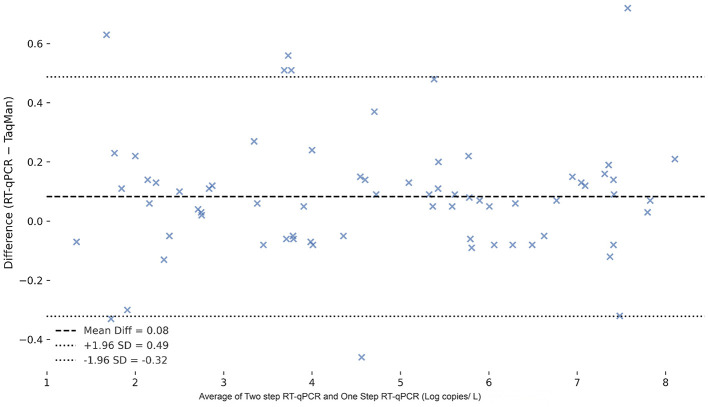
Bland-Altman plot comparing two-step qPCR and one-step qPCR methods for the detection of CAPRV in spleen tissue samples and water samples. The differences between Ct values obtained by the two methods are plotted against the mean Ct values. The red dashed line represents the mean difference (bias), and the green dashed lines indicate the 95% limits of agreement (mean ± 1.96 SD).

To assess the agreement between detection methods, a Bland–Altman analysis was performed comparing the TaqMan two-step qPCR and one-step qPCR results in spleen tissue samples (Figure 11). The Bland–Altman analysis demonstrated excellent agreement between the two-step qPCR and one-step qPCR assays, with a negligible mean bias of −0.021 and narrow limits of agreement (−0.174 to 0.132), indicating that the two methods yield highly consistent results across a range of concentrations.

#### 3.5.2 Copies of the virus in infected organs

**Figure 12:**
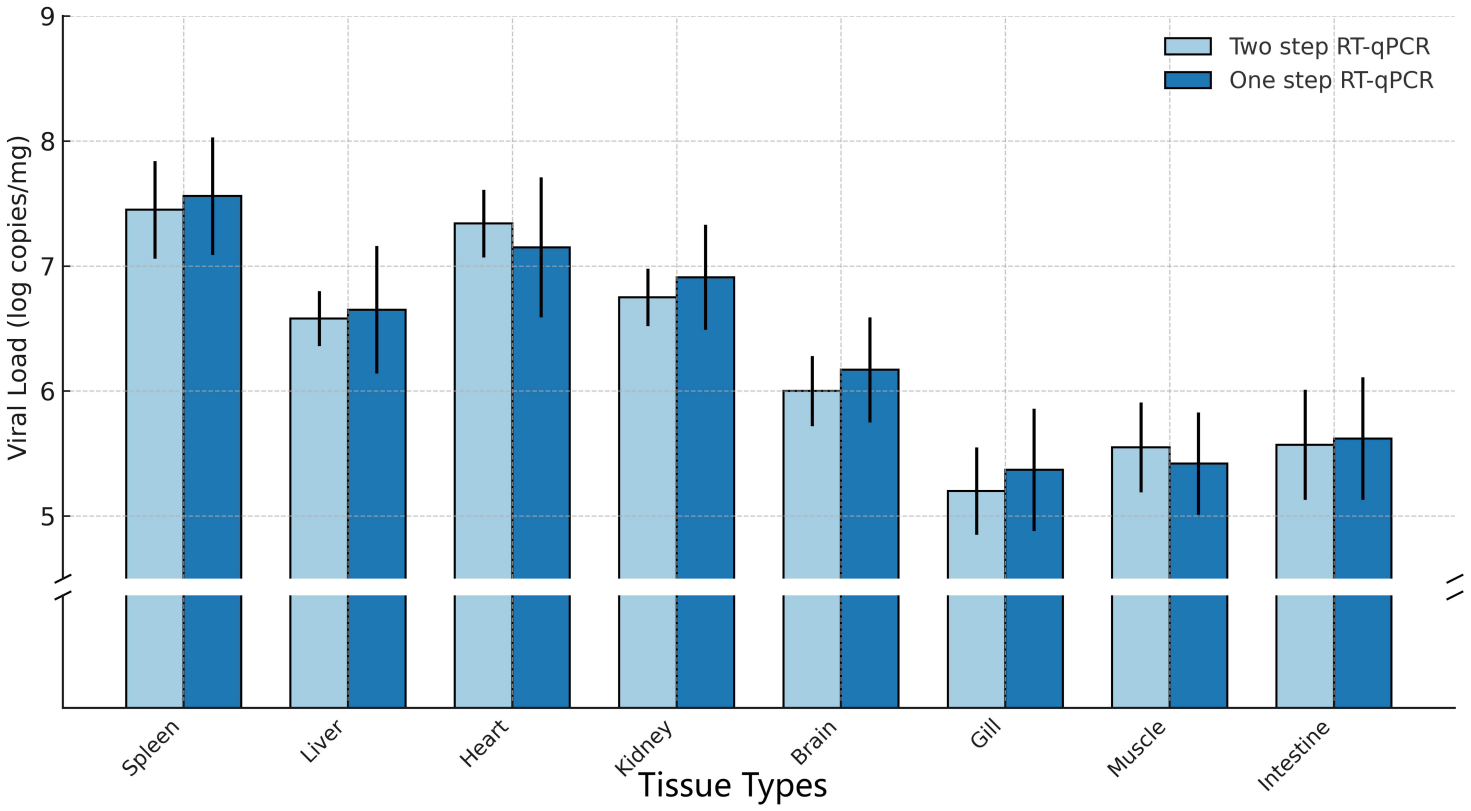
Viral copy numbers in different tissues of diseased fish using various qPCR Methods. Each bar represents the mean ± standard deviation (SD) of three independent replicates.

The two-step and one-step qPCR were used to study viral tissue distribution and identify the target organs of CAPRV2023 in golden pompano. In this study, the quantification of viral genome copy numbers (log copies/μg total RNA) using both two-step qPCR and one-step qPCR revealed notable differences across various tissues. As shown in Figure 12, viral loads were highest in the spleen (two-step qPCR: 10^7.45 ± 0.39^, one-step qPCR: 10^7.56 ± 0.47^) and liver (two-step qPCR: 10^6.58 ± 0.22^, one-step qPCR: 10^6.65 ± 0.51)^, followed by the heart (two-step qPCR:10^7.34 ± 0.27^, one-step qPCR: 10^7.15 ± 0.56^) and kidney (two-step qPCR:10^6.75 ± 0.23^, one-step qPCR:10^6.91 ± 0.42^). In contrast, lower viral genome copy numbers were found in the brain (two-step qPCR:10^6.00 ± 0.28^, one –step qPCR: 10^6.17 ± 0.42^), gill (two-step qPCR:10^5.20 ± 0.35^, one-step qPCR: 10^5.37 ± 0.49^), muscle (two-step qPCR:10^5.55 ± 0.36^, one-step qPCR:10^5.42 ± 0.41^) and intestine (two-step qPCR:10^5.57 ± 0.44^, one-step qPCR: 10^5.62 ± 0.49^). The significance analysis indicated that the viral genome copy numbers in all tissues did not differ significantly between the two detection assays (*p* > 0.4).

## 4 Discussion

Recently, a devastating epidemic associated with CAPRV2023 infection has caused substantial mortality in golden pompano populations across China, posing a serious threat to the sustainability of the aquaculture industry (2). To date, no effective preventive or therapeutic strategies have been established. Therefore, early and accurate detection of this emerging pathogen is essential for timely intervention and effective disease management. Existing detection methods, including conventional PCR and SYBR Green I-based qPCR assays developed by Sun et al. (2), require further validation in terms of specificity and sensitivity. In this study, we successfully developed a novel two-step qPCR assay and a one-step qPCR assay for CAPRV2023. Both assays demonstrated superior specificity, sensitivity, and reproducibility compared to previously reported techniques.

The G protein of fish rhabdoviruses plays a crucial role in membrane fusion and maintenance of virulence and is a key antigen that induces virus-neutralizing antibodies and host immunity (14). Owing to its high degree of conservation across various species of rhabdoviruses, the G protein gene serves as an ideal target for developing detection techniques (15, 16). The detection limit of the newly developed two-step qPCR assay for CAPRV2023 was comparable to that achieved for white spot syndrome virus using qPCR and lower than those for koi herpesvirus, VHSV, and infectious pancreatic necrosis virus (20–100 copies/μL). It was also 500-fold higher than the detection limit of 10^3^ copies/μL obtained using conventional PCR (17–19). Although the one-step qPCR showed slightly reduced sensitivity than the two-step qPCR assay targeting the same gene, it still outperformed most molecular diagnostic methods reported for aquatic viral pathogens. For instance, the detection limit of a one-step RT-LAMP assay for TiLV was ~100 copies/μL (20), and for nodavirus genotypes typically range between 50 and 100 copies/μL. These findings highlight the high analytical sensitivity and practical applicability of the developed the two qPCR assays for rapid and reliable detection of CAPRV2023.

During an extensive epidemiological investigation that spanned the entire farming cycle of golden pompano—lasting over 8 months and covering multiple geographic regions. The two-step and one-step qPCR assays demonstrated exceptional robustness. They consistently detected all CAPRV2023-positive samples, indicating high geographic adaptability and strong tolerance to potential viral mutations. Notably, regions such as Central Zhanjiang and South Zhanjiang exhibited elevated positivity levels in July and September. The presence of CAPRV2023-positive samples in Yangjiang during January, despite the typically lower environmental temperatures (17.5°C), deviates from previously documented seasonal patterns. This unexpected finding may suggest enhanced environmental adaptability of the virus, potentially due to genetic mutations that improve viral survival and transmissibility under colder conditions (21). Alternatively, localized aquaculture practices might provide favorable microenvironments for viral persistence. These findings highlight the necessity for comprehensive molecular and ecological studies to elucidate the mechanisms underlying seasonal variation.

From an epidemiological perspective, IHNV is recognized as being horizontally transmitted through mucus and sexual fluids released by infected fish (22). In a previous study, CAPRV2023 infection was successfully induced through exposure to contaminated water (2), highlighting the potential value of environmental surveillance. In this investigation, water samples consistently yielded higher detection rates across all methods, with the two-step and one-step qPCR again demonstrating their highest sensitivity. Moreover, methods such as ultrafiltration, as demonstrated by Othman et al. (23), have shown considerable promise in improving virus retention from water samples. Although the reported viral retention efficiency was relatively modest (54.03 ± 8.25%) (23), the method still contributes meaningfully to viral concentration and downstream molecular detection, particularly when combined with sensitive qPCR-based diagnostics. In our study, the ultrafiltration approach enabled efficient viral concentration, offering a rapid, cost-effective, and reusable solution for routine monitoring. Altogether, these findings support the feasibility of using environmental water samples for early warning of CAPRV2023 outbreaks, with the two qPCR assays serving as a highly sensitive and reliable diagnostic tools.

The one-step qPCR assay offers distinct advantages over the conventional two-step assay, particularly for large-scale disease monitoring in aquaculture. The two-step assay requires separate reverse transcription and amplification steps, which prolongs the process and increases the likelihood of contamination and inconsistent results due to repeated pipetting operations. These challenges are exacerbated when processing a high volume of samples or conducting analyses under field conditions with limited resources. Although the one-step qPCR shows slightly reduced sensitivity compared to the two-step assay, its operational simplicity, speed, and lower contamination risk make it especially suitable for field settings, where rapid decision-making and ease of use are critical. The one-step qPCR assay developed in this study addresses these limitations by integrating both reverse transcription and amplification into a single closed-tube procedure. This approach not only simplified the workflow but also minimizes handling time and reduces the risk of errors or exposure to environmental contaminants. Consequently, the one-step qPCR is particularly suitable for rapid and reliable testing during disease outbreaks, where efficient screening of numerous clinical or environmental samples is critical.

The primers, probes, and enzymes developed in this study have the potential to be formulated into lyophilized dry powder kits, greatly enhancing their stability and facilitating transportation. Combined with the increasing affordability and miniaturization of qPCR instruments, this assay shows significant promise for on-site aquaculture applications. Portable qPCR systems integrated with these ready-to-use kits can serve as a rapid and reliable diagnostic tool for frontline aquaculture practitioners to detect viruses early and monitor disease progression. This capability enables timely interventions to mitigate disease outbreaks, reduce losses, and improve farm management practices. The integration of advanced molecular diagnostic technologies into aquaculture workflows represents a transformative step forward in promoting sustainable disease management and fostering long-term industrial growth.

Beyond disease surveillance, the high sensitivity and adaptability of the developed qPCR assays offer valuable tools for studying CAPRV2023 pathogenesis. These assays can be applied to monitor viral load dynamics, investigate tissue tropism, and explore host–virus interactions during acute and persistent infections. Furthermore, their reliable performance enables targeted screening of broodstock, juvenile fish, and environmental reservoirs, contributing to more effective and proactive biosecurity protocols in aquaculture systems.

Although the present study demonstrates the high diagnostic performance of the one-step qPCR assay, several areas warrant further investigation. First, continuous genomic surveillance of circulating CAPRV strains is essential to monitor potential mutations that may affect primer or probe binding sites. Second, large-scale field validation across diverse farming conditions and additional host species will help assess the generalizability and robustness of the assay. Third, the integration of this method into portable detection platforms could facilitate rapid, on-site diagnosis, improving outbreak response times. Lastly, further exploration of environmental reservoirs and transmission dynamics may enhance early warning systems and contribute to the development of comprehensive disease management strategies in aquaculture systems.

## 5 Conclusion

The two-step and one-step qPCR assays developed in this study offer rapid, sensitive, and accurate molecular approach for detecting CAPRV2023 in field samples. These assays could serve as valuable tools for epidemiological studies, early disease warning, pathogenesis research, and disease prevention and control.

## Data Availability

The datasets presented in this study can be found in online repositories. The names of the repository/repositories and accession number(s) can be found in the article/Supplementary material.
