# Correction: One-step and two-step qPCR assays for CAPRV2023: development and application in full-cycle epidemiological surveillance of golden pompano

**DOI:** 10.3389/fvets.2026.1848905

**Published:** 2026-05-11

**Authors:** Heng Sun, Bissih Fred, Haoyu Wang, Jie Huang, Zihao Wu, Dandan Wu, Yishan Lu, Jichang Jian, Yucong Huang

**Affiliations:** 1 Guangdong Provincial Key Laboratory of Aquatic Animal Disease Control and Healthy Culture, Fisheries College of Guangdong Ocean University, Zhanjiang, China; 2 Key Laboratory of Control for Disease of Aquatic Animals of Guangdong Higher Education Institutes, Fisheries College, Guangdong Ocean University, Zhanjiang, China

**Keywords:** *Carpione rhabdovirus*, CAPRV2023, *Trachinotus ovatus*, TaqMan quantitative PCR, one-step qPCR, viruses in water samples

Affiliation “Guangdong Provincial Key Laboratory of Aquatic Animal Disease Control and Healthy Culture, Fisheries College of Guangdong Ocean University, Zhanjiang, China” was erroneously given as “Guangdong Provincial Key Laboratory of Aquatic Animal Disease Control and Healthy Culture, Zhanjiang, China.”

The original version of this article has been updated.

