# Supplementary material for: One-step and two-step qPCR assays for CAPRV2023: development and application in full-cycle epidemiological surveillance of golden pompano
Source: Front Vet Sci. 2025 Jul 31;12:1620997. doi: 10.3389/fvets.2025.1620997 (PMC12351644; doi:10.3389/fvets.2025.1620997)
Supplement: Supplementary file 1 [file Table_1.docx]

|  | | **Water Sample Detection** | | | **Spleen Tissue Sample Detection** | | |
| --- | --- | --- | --- | --- | --- | --- | --- |
| **Sample Collection Period** | **Region** | **Sample** | **Conventional**  **PCR** | **One step--qPCR (Log Copies/μL）** | **Conventional**  **PCR** | **One step--qPCR** | **Body length(cm)** |
| 2024.6 | Central Zhanjiang, Guangdong | 1-1 | + | 2.28 | + | 6.26 | 17.63 |
|  |  | 1-2 | + | 1.79 | + | 6.23 | 12.76 |
|  |  | 1-3 | + | 1.57 | + | 6.34 | 15.41 |
| 2024.6 | Beihai,  Guangxi | 2-1 | - | 0.62 | - | 1.61 | 18.44 |
|  |  | 2-2 | - | 0.68 | - | 1.78 | 17.63 |
|  |  | 2-3 | - | 0.74 | - | 1.93 | 14.12 |
| 2024.6 | Xuwen,Zhanjiang  Guangdong | 3-1 | - | 0.67 | - | 2.15 | 19.32 |
|  |  | 3-2 | - | 0.76 | - | 1.89 | 16.44 |
|  |  | 3-3 | - | 0.89 | - | 1.62 | 18.10 |
| 2024.7 | Leizhou,Zhanjiang,Guangdong | 4-1 | + | 2.47 | + | 6.37 | 15.47 |
|  |  | 4-2 | + | 2.26 | + | 7.23 | 16.38 |
|  |  | 4-3 | + | 2.78 | + | 6.78 | 15.84 |
| 2024.7 | Yangjiang,  Guangdong | 5-1 | None | None | - | - | None |
|  |  | 5-2 | None | None | - | - | None |
|  |  | 5-3 | None | None | - | - | None |
| 2024.7 | Xuwen,Zhanjiang  Guangdong | 6-1 | - | 1.19 | + | 4.34 | 14.32 |
|  |  | 6-2 | - | 1.43 | + | 4.86 | 15.57 |
|  |  | 6-3 | - | 1.35 | + | 4.56 | 14.48 |
| 2024.7 | Qinzhou  Guangxi | 7-1 | None | None | + | 3.68 | None |
|  |  | 7-2 | None | None | + | 3.47 | None |
|  |  | 7-3 | None | None | + | 4.28 | None |
| 2024.7 | Central Zhanjiang,Guangdong | 8-1 | + | 2.47 | + | 6.43 | 20.23 |
|  |  | 8-2 | + | 2.65 | + | 6.79 | 17.81 |
|  |  | 8-3 | + | 2.18 | + | 5.87 | 19.36 |
| 2024.8 | Leizhou,Zhanjiang,Guangdong | 9-1 | - | 1.44 | + | 4.82 | 18.70 |
|  |  | 9-2 | + | 1.81 | + | 5.53 | 16.37 |
|  |  | 9-3 | - | 1.38 | + | 6.03 | 19.88 |
| 2024.8 | Yangjiang,  Guangdong | 10-1 | - | 1.79 | + | 4.85 | 19.36 |
|  |  | 10-2 | - | 1.93 | + | 4.74 | 20.73 |
|  |  | 10-3 | - | 2.1 | + | 4.98 | 21.65 |
| 2024.8 | Hainan province | 11-1 | None | None | - | 1.30 | None |
|  |  | 11-2 | None | None | - | 0.85 | None |
|  |  | 11-3 | None | None | - | - | None |
| 2024.9 | Leizhou,Zhanjiang,Guangdong | 12-1 | - | 1.56 | + | 6.45 | 22.13 |
|  |  | 12-2 | + | 2.36 | + | 6.73 | 23.74 |
|  |  | 12-3 | + | 2.13 | + | 5.98 | 24.60 |
| 2024.9 | Yangjiang,  Guangdong | 13-1 | None | None | + | 2.49 | 27.64 |
|  |  | 13-2 | None | None | + | 2.35 | 28.05 |
| 2024.9 | Techeng Zhanjiang,Guangdong | 14-1 | - | - | - | - | 25.23 |
|  |  | 14-2 | - | - | - | - | 26.44 |
|  |  | 14-3 | - | - | - | - | 25.58 |
|  |  | 14-4 | - | - | - | - | 24.26 |
| 2024.9 | Central Zhanjiang,Guangdong | 15-1 | - | 1.06 | + | 4.37 | 27.96 |
|  |  | 15-2 | - | 1.64 | + | 5.65 | 29.02 |
|  |  | 15-3 | - | 1.55 | + | 4.66 | 27.55 |
|  |  | 15-4 | - | 1.11 | + | 5.27 | 28.40 |
| 2024.10 | Techeng Zhanjiang,  Guangdong | 16-1 | - | - | - | - | 23.31 |
|  |  | 16-2 | - | - | - | - | 24.44 |
|  |  | 16-3 | - | 1.47 | - | - | 26.50 |
|  |  | 16-4 | - | - | - | - | 28.07 |
|  |  | 16-5 | - | 1.87 | + | 2.88 | 24.29 |
|  |  | 16-6 | - | 1.75 | + | 3.05 | 23.74 |
| 2024.10 | Nansan  Zhanjiang,  Guangdong | 17-1 | - | 1.26 | - | 1.95 | 29.55 |
|  |  | 17-2 | - | 1.17 | - | 2.16 | 30.17 |
|  |  | 17-3 | - | - | - | - | 29.38 |
|  |  | 17-4 | - | - | - | - | 27.94 |
| 2024.10 | Techeng Zhanjiang,  Guangdong | 18-1 | - | 1.33 | - | 1.13 | 28.60 |
|  |  | 18.2 | - | 0.96 | - | 1.69 | 24.76 |
| 2024.10 | Techeng Zhanjiang,  Guangdong | 19-1 | - | 1.72 | - | - | 25.30 |
|  |  | 19-2 | - | 1.23 | - | - | 9.12 |
|  |  | 19-3 | - | 1.38 | - | - | 22.54 |
|  |  | 19-4 | - | 1.25 | - | - | 25.78 |
| 2024.11 | Techeng Zhanjiang,  Guangdong | 20-1 | - | 1.51 | - | 2.54 | 29.45 |
|  |  | 20-2 | - | 1.15 | - | 2.82 | 27.89 |
| 2024.11 | Caotan Zhanjiang,  Guangdong | 21-1 | - | - | - | - | 23.96 |
|  |  | 21-2 | - | - | - | - | 24.65 |
|  |  | 21-3 | - | - | - | - | 28.11 |
|  |  | 21-4 | - | - | - | - | 26.38 |
| 2024.11 | Techeng Zhanjiang,  Guangdong | 22-1 | - | 1.23 | - | 2.78 | 30.05 |
|  |  | 22-2 | - | 1.41 | - | 2.22 | 29.34 |
|  |  | 22-3 | - | 1.37 | - | 2.69 | 27.03 |
| 2024.11 | Nansan Zhanjiang,  Guangdong | 23-1 | - | - | - | - | 21.14 |
|  |  | 23-2 | - | 0.92 | - | 1.37 | 22.46 |
|  |  | 23-3 | - | - | - | - | 18.23 |
| 2024.12 | Central Zhanjiang,  Guangdong | 23-1 | - | - | - | - | 45.29 |
|  |  | 23-2 | - | 1.08 | - | - | 40.77 |
|  |  | 23-3 | - | 0.76 | - | - | 41.58 |
| 2024.12 | Nansan Zhanjiang,  Guangdong | 23-1 | - | - | - | - | 23.69 |
|  |  | 23-2 | - | 0.79 | - | - | 24.23 |
|  |  | 23-3 | - | 0.62 | - | - | 26.67 |
| 2024.12 | Leizhou Zhanjiang,  Guangdong | 24-1 | - | 1.48 | - | 4.03 | 17.67 |
|  |  | 24-2 | + | 2.16 | - | 4.57 | 16.94 |
| 2024.12 | Techeng Zhanjiang,  Guangdong | 25-1 | - | 0.67 | - | 1.26 | 8.23 |
|  |  | 25-2 | - | 1.19 | - | 1.79 | 7.54 |
|  |  | 25-3 | - | 0.86 | - | 1.57 | 9.76 |
| 2024.12 | Leizhou Zhanjiang,  Guangdong | 26-1 | - | 1.76 | - | 3.02 | 18.23 |
|  |  | 26-2 | - | 1.41 | - | 3.81 | 19.17 |
| 2025.1 | Yangjiang,  Guangdong | 27-1 | None | None | + | 5.1 | None |
|  |  | 27-2 | None | None | + | 5.31 | None |
| Positive  rate | | | 19.4% | 77.63% | 41.4% | 66.2% |  |

Note: "+" indicates positive results, "-" indicates negative results, and "None" denotes the absence of measurement data.
